# Supplementary material for: Novel Driver Strength Index highlights important cancer genes in TCGA PanCanAtlas patients
Source: PeerJ. 2022 Aug 11;10:e13860. doi: 10.7717/peerj.13860 (PMC9375969; doi:10.7717/peerj.13860)
Supplement: Supplemental Information 2 [file peerj-10-13860-s002.zip › COHORTS/genes plots/detailed/2021_8_16_14_9_distribution_events_detailed_females_26.pdf]

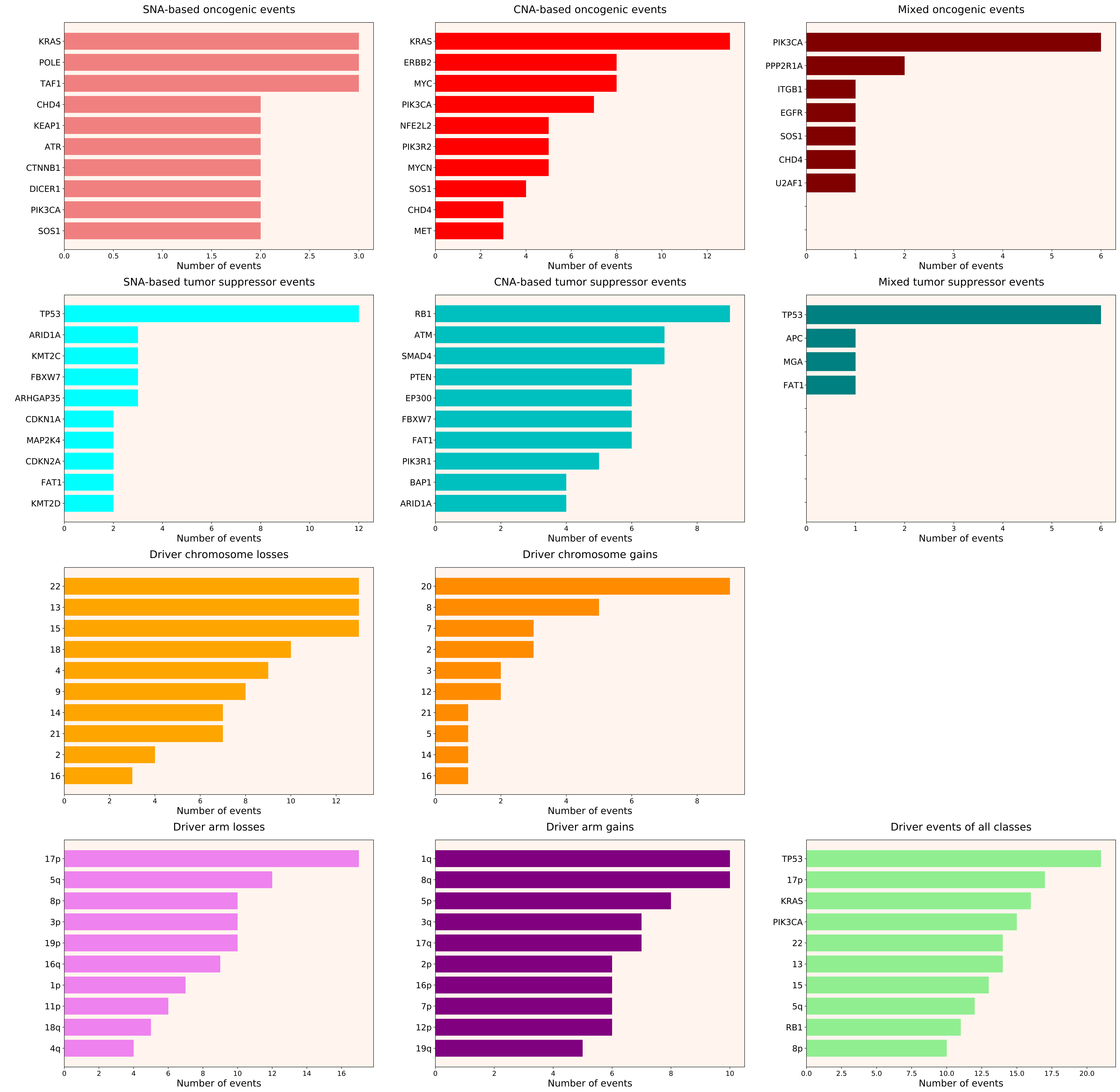
SNA-based oncogenic events
KRAS
POLE
TAF1
CHD4
KEAP1
ATR
CTNNB1
DICER1
PIK3CA
SOS1
Number of events
CNA-based oncogenic events
KRAS
ERBB2
MYC
PIK3CA
NFE2L2
PIK3R2
MYCN
SOS1
CHD4
MET
Number of events
Mixed oncogenic events
PIK3CA
PPP2R1A
ITGB1
EGFR
SOS1
CHD4
U2AF1
Number of events
SNA-based tumor suppressor events
TP53
ARID1A
KMT2C
FBXW7
ARHGAP35
CDKN1A
MAP2K4
CDKN2A
FAT1
KMT2D
Number of events
CNA-based tumor suppressor events
RB1
ATM
SMAD4
PTEN
EP300
FBXW7
FAT1
PIK3R1
BAP1
ARID1A
Number of events
Mixed tumor suppressor events
TP53
APC
MGA
FAT1
Number of events
Driver chromosome losses
22
13
15
18
4
9
14
21
2
16
Number of events
Driver chromosome gains
20
8
7
2
3
12
21
5
14
16
Number of events
Driver arm losses
17p
5q
8p
3p
19p
16q
1p
11p
18q
4q
Number of events
Driver arm gains
1q
8q
5p
3q
17q
2p
16p
7p
12p
19q
Number of events
Driver events of all classes
TP53
17p
KRAS
PIK3CA
22
13
15
5q
RB1
8p
Number of events
